# Behavioral Causes, Ecological Consequences, and Management Challenges Associated with Wildlife Foraging in Human-Modified Landscapes

**DOI:** 10.1093/biosci/biaa129

**Published:** 2020-11-11

**Authors:** Gaelle Fehlmann, M Justin O'riain, Ines FÜrtbauer, Andrew J King

**Affiliations:** Max Planck Institute for Animal Behavior, Bodensee, Germany; Institute for Communities and Wildlife, Africa, Department of Biological Sciences, University of Cape Town, Cape Town, South Africa; Behavioural Ecology and Endocrinology Laboratory and Andrew King is an associate professor and head of the SHOAL group in the Department of Biosciences at Swansea University, Swansea, Wales, United Kingdom; Max Planck Institute for Animal Behavior, Bodensee, Germany

**Keywords:** human–wildlife conflict, raiding, behavioral plasticity, movement ecology, time and energy budgets

## Abstract

Humans have altered up to half of the world's land surface. Wildlife living within or close to these human-modified landscapes are presented with opportunities and risks associated with feeding on human-derived foods (e.g., agricultural crops and food waste). Understanding whether and how wildlife adapts to these landscapes is a major challenge, with thousands of studies published on the topic over the past 10 years. In the present article, we build on established theoretical frameworks to understand the behavioral causes of crop and urban foraging by wildlife. We then develop and extend this framework to describe the multifaceted ecological consequences of crop and urban foraging for the individuals and populations in which they arise, with emphasis on social species for which interactions with people are, on balance, negative (commonly referred to as raiding species). Finally, we discuss the management challenges faced by urban and rural land managers, businesses, and government organizations in mitigating human–wildlife conflicts and propose ways to improve the lives of both wildlife and humans living in human-modified landscapes and to promote coexistence.

Up to 50% of the Earth's land surface has been modified by human activities, with 12% dedicated to crops (Ramankutty et al. 2008) and nearly 1% to cities (Liu et al. 2014). Natural environments are shrinking, and transition zones between natural and human-modified spaces are eroding, resulting in increased contact between humans and wildlife (Woodroffe and Ginsberg 1998). Wildlife species living within or close to a human-modified landscape typically experience a drastic change in resource availability, especially food. Traditional food resources can become depleted or destroyed, and agricultural crops or human and pet foods offer potentially novel food sources. Such human-derived foods can be rich in energy and predictable in time and space (Griffin et al. 2017), making crop foraging (table 1; Naughton Treves 1998), or urban foraging (Sol et al. 2013, Barrett et al. 2018, Santini et al. 2019), sometimes known as *raiding* (table 1), a highly rewarding foraging strategy for wildlife.

**Table 1.** Definition of terms.

Term	Definition	References
Behavioral plasticity	Plasticity is usually defined as innate phenotypic plasticity that can depend on genetic factors resulting in a constant behavioral trait that can vary across individuals, populations or species. Developmental plasticity refers to learning procedures, in which an animal can learn adaptive behaviors by trial and errors or cognitive abilities when exposed to a new situation.	Snell-Rood 2013
Crop foraging	Entering agricultural landscape in order to consume crops. In the present article, we frequently associate crop foraging with livestock depredation by wild carnivores. It is however distinct from crop damage resulting from moving through the agricultural landscape, which can have distinct causes and consequences on animals’ biology.	Naughton Treves 1998, Davies et al. 2011, Hill 2017
Human–wildlife conflict	Negative interactions between humans and wildlife. For example, disease transmission between humans and wildlife, raiding behavior by wildlife	
(crop-foraging or urban-foraging), physical aggression between humans and wildlife.	Donnelly et al. 2006, Acharya et al. 2016, Liu et al. 2011, Woodroffe et al. 2005	
Raiding behavior	Raiding manifests in wildlife entering human landscapes or directly interacting with humans in order to access human food sources. Elephants, primates, wild felids and bears are among the most high profile problematic species, but raiding does manifest in other genus. Throughout the present article, we minimize our use of the term raiding and, instead, favor crop foraging or urban foraging.	Hockings and McLennan 2015,
Lewis et al. 2015, Thouless and Sakwa 1995, Zarco-Gonzalez and Monroy-Vilchis 2014		
Socioecology	Study of social behavior and dynamics (e.g., cohesion, leadership) with regard to species ecology (e.g., predation risks, food availability).	Jarman 1974, Linklater 2000, Wrangham et al. 1993
Movement ecology	Study of the general movement of an animal resulting in patterns of occupation and landscape use, represented by features such as habitat selection or home ranges.	Börger et al. 2006, Burt 1943, Matthiopoulos 2003
Time and energy budgets	The two main budgets or currencies animals have to balance in order to maintain their body condition and reproduce. This notion is used in optimal foraging theories dividing species into time minimizers (species gaining fitness by limiting the time dedicated to foraging such as carnivores) and energy maximizers (species gaining fitness by increases energy intake such as elephants)	Charnov 1976, Hixon 1982,
Stephens and Krebs 1986		
Urban foraging	Urban foraging is broad term to refer to any foraging event happening in the urban or any built environments (residential or commercial property). Food items can be as varied as seeds from bird feeders or people voluntarily feeding wildlife, plant materials from gardens, parks and street trees, food discards from litter bins, food items from restaurants, residential or commercial properties, pets.	Sol et al. 2013, Santini et al. 2019

Human–wildlife interactions can bring positive effects for both humans and wildlife, enhancing human well-being (Chan et al. 2007), with a famous example being bird feeding in urban environments (Reynolds et al. 2017). However, when wildlife and humans compete for the same resources or space, conflicts may arise (Bruskotter et al. 2017). Indeed, crop foraging by wildlife can negatively affect local economies (Chan et al. 2007). For example, in rural Uganda, elephants (*Loxodonta africana*) can damage up to 6510 square meters of crops in a single foraging trip (Naughton Treves 1998). In the urban environment, gulls (*Larus* spp.) take food directly from people across the globe (Spelt et al. 2019), black bears (*Ursus americanus*) in North America enter urban environments to forage on garbage (Lewis et al. 2015), and, in Asia, macaques (*Macaca fascicularis*) enter and damage houses and commercial properties to access human foods (Yeo and Neo 2010). When opportunistic crop and urban foraging by wildlife is not tolerated by people (Carter and Linnell 2016, Bruskotter et al. 2017), this results in conflict with people that compromises both human and wildlife well-being (Barua et al. 2012, Hill 2017).

Studies of animal behavior are increasingly adopting an integrated ecological approach (Nathan et al. 2008) and strive for *in situ* quantitative studies (King et al. 2018) that link animals’ behavior to the complex environments in which they live, including human-altered landscapes (Caro 1999). Empowered by recent technological (Fehlmann and King 2016), statistical (Franz and Nunn 2009, Koen et al. 2014, Williams et al. 2020), and conceptual (Dingemanse et al. 2010, Sih 2013, Gallagher et al. 2017) advances, the last decade has seen a growing body of literature emphasizing the importance of anthropogenic factors on species’ biology (Tuomainen and Candolin 2011, Sih 2013, Sol et al. 2013, Fleming and Bateman 2018, Santini et al. 2019). However, in only a few studies have addressed the multifaceted aspects of wildlife behavioral adaptations and linked them to the conflicts that can emerge from this adaptability. In the present article, we adopt a behavioral ecology approach to understand crop and urban foraging and discuss how such knowledge is necessary for achieving human coexistence with wildlife when conflicts arise. We apply these established theoretical and statistical frameworks to first understand the behavioral causes of crop and urban foraging by wildlife, describing how and why some individuals (and species) cope better with human-induced changes to their environment (Sih 2013, Sol et al. 2013, Santini et al. 2019). Then, we build on the general approach developed in Tuomainen and Candolin (2011) and review disparate studies in various contexts to define the multifaceted ecological consequences of crop and urban foraging for the spatial and socioecology of terrestrial species. In this section, we mainly focus on species that are typically in conflict with humans over food resources (commonly named *raiding* species) with a particular emphasis on social species. Finally, we end by discussing a route for mitigating human–wildlife conflict (table 1) and solving some of the management challenges associated with animals foraging in a human-altered landscape. We believe that taking and applying a behavioral ecology approach is key to mitigating human wildlife conflicts in various contexts, reducing its severe consequences on both human and wildlife well-being (Barua et al. 2012, Hill 2017).

## The behavioral causes of wildlife foraging in human-modified landscapes

Wildlife exploiting human-derived resources is not new, but a growing number of species have been reported to be colonizing or recolonizing human-altered environments in recent years (Bruskotter et al. 2017). A key challenge is to determine why and how some species and individuals thrive in human-altered environments, whereas others fail. This challenge has been the subject of recent reviews, frameworks, and syntheses (e.g., Tuomainen and Candolin 2011, Sih 2013, Griffin et al. 2017, Santini et al. 2019) that indicate that surviving and reproducing in a dynamic, human-modified environment require a flexible phenotype that alters to match the current environment (Sol et al. 2002, Wright et al. 2010). Flexibility has therefore become central to understanding a species's or individual's responses to altered environments and whether they exploit new food resources. Indeed, although human-derived foods tend to be high in calories and to have short handling times (Griffin et al. 2017, Hill 2017), their inclusion in the diets of species that have access to these resources is not a given (Sih 2013, Barrett et al. 2018).

At a species level, the degree of dietary specialization and niche overlap with human-altered environments can be useful predictors of success in exploiting novel foods. Generalist species that can exploit a wide spectrum of food items and habitats are better at recognizing potential risks and exploiting opportunities associated with human-altered landscapes and are therefore among the first to settle in these environments (Colles et al. 2009, Sih 2013, Santini et al. 2019). However, even specialists can thrive where species are preadapted to human-altered environments (Griffin et al. 2017). For instance, raptor species that nest on cliffs are perfectly suited to exploit high-rise buildings in cities, where they can prey on abundant populations of urban pigeons (generalists) that thrive in such landscapes (Chace and Walsh 2006). The specialist raptor species exploiting high-rise buildings is a good example of where the human-changed landscapes fit within a species's fundamental niche (MacMahon et al. 1981). The overlap between the fundamental niche and the human-altered landscape is therefore another predictor of the likelihood of a given species to use this novel space. In contrast, where species fundamentally change their behavior so that they are able to exploit human-derived resources and landscapes, this requires that they expand their fundamental niche (MacMahon et al. 1981). Indeed, to exploit human-derived resources that are entirely novel requires behavioral flexibility, a trait that characterizes generalist species (Daniels et al. 2019).

The flexibility that characterizes the behavior of generalist species can also help explain within-species differences (interindividual differences) in crop or urban foraging (Wright et al. 2010, Snell-Rood 2013). Interindividual differences in sex, age, size, and personality may determine the propensity (or ability) to forage in human-modified landscapes (Camphuysen et al. 2015, Ducatez et al. 2017, Brooks et al. 2020). For example, sex- and age-specific life history trade-offs are correlated with behavioral risk aversion across species and contexts (see Smith and Blumstein 2008 for a meta-analysis) and may explain why males and older individuals often forage in human spaces more frequently than females and younger individuals (Strum 2010, Chiyo et al. 2012).

**Figure 1.**
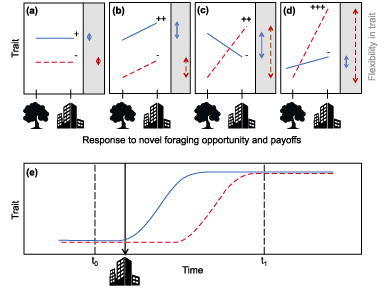
Interindividual differences in responses to a novel foraging opportunity. Behavioral trait expressed by two individuals (plain and dashed lines) before and after exposure to a human-altered landscape. Where the behavior allows an individual to benefit from the human-modified landscape (e.g., via crop or urban foraging), this is indicated by a plus sign; where behaviors result in costs for individuals (e.g., they are unable to access human-derived foods) or an increased risk and cost of injury, they are indicated by a hyphen. The grey box to the right of each panel illustrates the degree of flexibility exhibited by each individual. (a) No flexibility by either individual, but one individual's behavior results in a net benefit and the other does not. (b) Similar flexibility for both individuals, but only one individual benefits because of the different intercept. (c) Between-individual variation in flexibility, correlated with the absolute level of traits; one individual benefits and one incurs a cost. (d) Between-individual variation flexibility, independent of the absolute value of traits; one individual benefits and one incurs a cost. (e) The level of a behavioral trait measured in two individuals at two time points, prior to (t_0_) and following human changes to the landscape (t_1_). The rate at which the two individuals react to the change differs, with the individual represented by the solid line reacting more quickly in this scenario.

Statistically, behavioral plasticity (table 1) may be explained as the slope of an individual's response to some change in environment (when behavior and change are plotted against one another). If an individual's phenotype does not change (i.e., the slope's coefficient is null; figure 1a), the individual is interpreted as not exhibiting flexibility. Where behavior is not flexible but where interindividual differences in behavior do exist (figure 1a), certain behavioral types may show a higher propensity to persist and forage in human-modified landscapes. Where an individual's phenotypes are altered in response to changing environments (i.e., the slope's coefficient is nonnull; figure 1b) and where individuals vary in their type of flexibility (figures 1c and 1d), those individuals whose change in behavior results in novel foraging strategies will be selected for (where this results in net benefits). Furthermore, those individuals that respond quickly (figure 1e) to changes (and in the correct way) should be especially good at exploiting new resources. Several detailed reviews provide in-depth accounts of how to study interindividual differences in personality and plasticity using the statistical and theoretical framework we have described above (Dingemanse et al. 2010, Wright et al. 2010, Snell-Rood 2013).

Learning is also crucial. Over the course of a lifetime, naive individuals can learn to recognize both opportunities and risks presented by human-modified landscapes (Snell-Rood 2013, Sol et al. 2013), and those individuals and populations most frequently exposed to new environments will be the fastest to develop novel foraging strategies by simple trial and error learning (e.g., Ducatez et al. 2013). In particular, species with long life spans and large home ranges will be more likely to experience and learn to exploit human-modified landscapes through their lifetime (e.g., African elephants, *Loxodonta africana*; Graham et al. 2009). Long-lived, wide-ranging species also tend to have impressive cognitive skills (e.g., Lefebvre et al. 2004), affording navigation in geographically complex environments where resources and risks are dictated by artificial human activity patterns (Griffin et al. 2017). Such skills also allow individuals that forage in agricultural and urban environments to properly assess and update risks that are related to specific locations and specific people (Sol et al. 2013, Bruskotter et al. 2017, Fehlmann et al. 2017b).

Therefore, the discovery of new resources may be challenging, and the number of niches an individual can exploit in the urban environment may be linked to its cognitive abilities. Moreover, if individuals live in social groups with frequent and repeated interactions, novel behaviors can become commonplace via social enhancement (e.g., Aplin et al. 2012), horizontal transmission (i.e., from group mates, e.g., Chiyo et al. 2012), or vertical transmission (i.e., parent to offspring, e.g., Mazur and Seher 2008). Social learning can therefore accentuate or accelerate the new behaviors within a population that allow individuals to derive benefits from urban or crop foraging. The chacma baboon (*Papio ursinus*) provides an excellent example of a behaviorally flexible, long-lived, wide-ranging, socially and cognitively complex species that thrives in human-modified landscapes (box 1).

**Box 1. Raiders of the human realm: Chacma baboons (*Papio ursinus*).**

Baboons, like other long-lived, social species, are well equipped to exploit opportunities presented by human-altered landscapes. First, manual dexterity, agility, and climbing ability allow baboons to enter human landscapes and get access to food items (Hoffman and O'Riain 2012b). Second, large brain size (which is correlated with sociality) may promote innovative behavior (Reader et al. 2011). Third, long life spans provide the opportunity for individual learning and social learning via multiple, long-term individualized social relationships (King et al. 2008). Fourth, overlapping generations allow novel behaviors to be transmitted from older to younger conspecifics more readily (Pereira 1995). Across Africa, baboons are notorious crop and urban foragers. Human-modified landscapes provide baboons the opportunity to forage on high-calorie human crops, foods and waste (Strum 2010). On the Cape Peninsula in the Western Cape Province of South Africa, 1 square kilometer of human-modified habitat (pine plantations and vineyards) can support nearly five times the number of baboons as the same area of natural habitat (Hoffman and O'Riain 2012a). These Cape baboons exploit spaces at the periphery of the city (e.g., vineyards) that are close to refuges (Fehlmann et al. 2017b), resting at the urban edge and waiting for suitable opportunities to exploit the resources in human-modified landscapes, engaging in brief, high-activity raids (Fehlmann et al. 2017a). These altered foraging dynamics result in smaller home ranges than groups elsewhere in the species range, which raid less often or not at all (Altmann and Muruthi 1988, Strum 2010, Hoffman and O'Riain 2012b), and directly alters the Cape baboon time and energy budgets (Doorn et al. 2010, Fehlmann et al. 2017a). High-energy food items and a relaxed time budget in crop- and urban-foraging baboons affords more time resting and improved body condition, which, ultimately is linked to higher biological fitness (Strum 2010). However, as for other primates (Hockings et al. 2015), conflicts with humans can result in severe injuries or death (Beamish 2009) or lead to culling and removal of individuals through management practices (Swan et al. 2017), which can have consequences for population size and stability (Beamish 2009).

**Figure:**
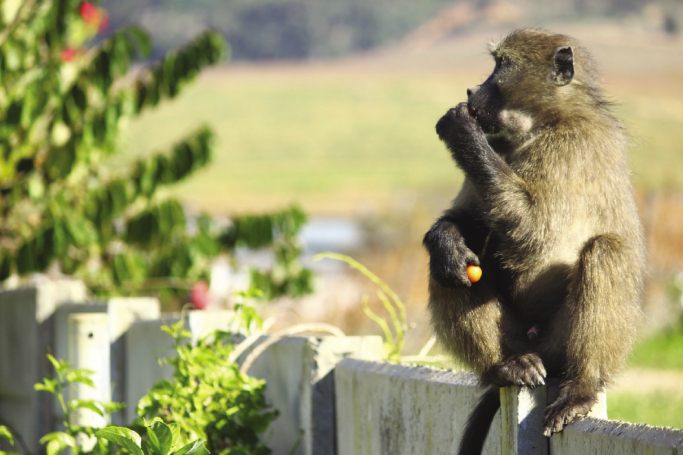
Papio ursinus. Photograph: Gaelle Fehlmann.

The theoretical and statistical frameworks for describing why some species and individuals are better able to exploit and to cope with human-induced changes to the environment are therefore well established and describe the overall causes of foraging in human-altered environments (Sol et al. 2002, Sih 2013, Barrett et al. 2018). This provides an excellent platform for understanding crop and urban foraging from both a mechanistic and functional (evolutionary perspective). However, we lack a comparable framework for describing the ecological consequences of crop and urban foraging for the individuals and populations in which they arise.

## The ecological consequences of wildlife foraging in human-modified landscapes

**Figure 2.**
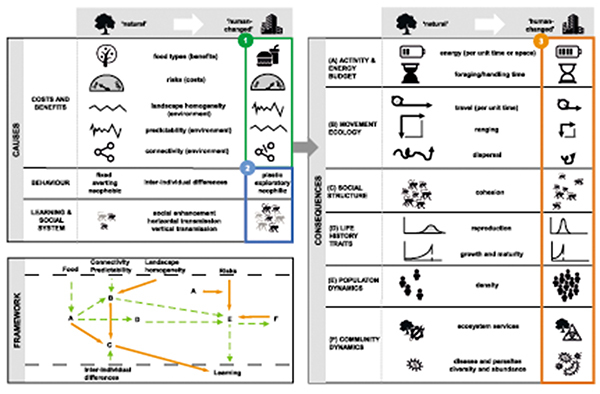
The framework of the ecological causes and consequences of foraging in a human-modified landscape. Environmental (1) and behavioral (2) factors affecting habitat choice at a species level are represented in the box labeled Causes. For individuals choosing to exploit anthropogenic resources, these causes have been shown to affect (A) activity and energy budgets, (B) movement ecology, (C) social structure, (D) life history traits, (E) population dynamics, and (F) community dynamics (see the box labeled “Consequences”). The interconnections between each effect (3) are represented too (see the box labeled “Framework”). Positive effects are represented with dashed arrows, and negative effects are show with plain arrows. For example, the increased energy consumed per unit of time and space and the associated diminished foraging and handling times (A) favor the reduction of the travelling time, decreas home range and the dispersal (B), and alter cohesion of social groups (D). Increased food quality also speeds growth and maturation, and increases reproduction rates (D). In turn, this results in higher population densities (E). These can favor biohazards (e.g., spread of diseases, parasites) and reduce ecosystem services (F).

In this section of the article, we focus on the ecological consequences of foraging in human-modified landscapes for terrestrial, mainly social species. In particular, we explore the consequences of foraging in human-modified landscapes for activity and energy budgets (table 1), because these are fundamentally linked to species’ foraging ecologies and are therefore predicted to be altered; movement ecology (table 1), because crop and urban foraging requires the exploitation of new landscape features; socioecological dynamics, which can be affected by interindividual variation in space use and propensity to exploit these new foraging opportunities; life history traits, which are likely to be affected as a result of the changes in the first three aspects; and, finally, population dynamics and community ecology, which emerge from the interactions of the first four categories. Although most of these aspects can be relevant for any species foraging in human-altered environments, we mostly build our discussion on those most reported in conflict with humans, which tend to be long-lived social species (Redpath et al. 2013). Descriptions and the relationships among these major factors with respect to foraging in human-modified landscapes are summarized in figure 2.

### Activity and energy budgets

Crop and urban foraging strategies have the potential to strongly affect a species's time and energy budgets, because these individuals generally exploit high-energy food items. For some species that require a fixed amount of energy in a day (e.g., time minimizers; Schoener 1971), this means they reach their energetic needs more quickly and with more certainty because of the predictability of food distribution (Fedriani et al. 2001, Beckmann and Berger 2003a). Chacma baboons, for example, are able to massively reduce the time they spend seeking food by foraging for crops or in urban environments (Hoffman and O'Riain 2012a, Fehlmann et al. 2017a). The time released by reduced foraging effort can then be reinvested in other activities, such as vigilance or resting (e.g., Altmann and Muruthi 1988, Fehlmann et al. 2017a) or social interactions such as grooming (e.g., Altmann and Muruthi 1988). The first can contribute to risk mitigation strategies (Roberts 1996, Beckmann and Berger 2003a, Lima et al. 2005), and the second can alleviate stress (Shutt et al. 2007, Hostinar et al. 2014, Wittig et al. 2016), which can be higher for individuals encountering humans (Ahlering et al. 2011, Fourie et al. 2015). However, when human-derived resources are distant from other vital resources (e.g., refuges, water), individuals and groups may actually have to increase their travel times and reduce their time resting in order to exploit these resources (Isaksson et al. 2016, Hill 2017, Enners et al. 2018). Overall, such changes in time and energy budgets can be viewed as a reflection of the differences in availability or quality between natural food items and crop- or urban-foraging options (Spelt et al. 2019).

### Movement ecology

Individuals that engage in crop or urban foraging will inevitably range across human-modified landscapes. Although such overlap may be the consequence of human encroachment into the existing home range of wild populations, in several cases, wildlife populations actively alter their space use to exploit new opportunities in human-dominated landscapes (Beckmann and Berger 2003a, Reher et al. 2016, Seiler and Robbins 2016).

Burt (1943) referred to the home range as “the area over which the animal normally travels in search of food.” In human-modified landscapes, species can fulfill their energetic needs faster, reducing the time and effort devoted to both the active and sedentary phases of foraging (Bartumeus et al. 2010, Šálek et al. 2015, Fehlmann et al. 2017a). In addition, crop-foraging species face highly homogeneous landscapes with largely predictable resources (Naughton Treves 1998) that may limit the need for exploration. As a result, the daily path length and home ranges for crop-foraging individuals and groups are typically reduced compared with wild foraging conspecifics (Davison et al. 2009, Bartumeus et al. 2010, Reher et al. 2016). For example, in yellow napped Amazons (*Amazona auropalliata*), birds foraging in croplands have a home range ten times smaller than birds foraging in pastures (Salinas-Melgoza et al. 2013). Similarly, many resources in the urban environment are at least predictable in space (e.g., refuse bins, bird feeders, restaurants), which can be highly attractive, especially for carnivores (Fleming and Bateman 2018). For example, hyenas (*Crocuta crocuta*) living next to human lodges in the Kruger National Park, in South Africa, focus their foraging efforts in specific areas and have smaller home range sizes than do groups elsewhere in the species range (Belton et al. 2016).

When individuals begin to forage on crops or in urban spaces, not only does the space they use change, but also what they do in this space. What behavior is performed and where is influenced by landscape attributes (e.g., topography, homogeneity, fragmentation; Shepard et al. 2013, Koen et al. 2014), or species interactions (e.g., predation risks; Fraser and Huntingford 1986). Human-modified areas have, for the most part, been subjected to rapid and fundamental changes in their structure. This includes changes in their topography (via earthmoving works such as for roads, agriculture or via urbanization; e.g., Fleming and Bateman 2018), substrate (from biochemical modifications in fields via fertilization to soil surface changes via concreting), and ecosystems (involving species eradication, displacement or the addition of exogenous species; e.g., Chace and Walsh 2006). Overall, this results in profound changes in connectivity of the environment and affects the costs and benefits associated with crop or urban foraging.

The different barriers animals can encounter in human-altered landscapes are equally likely to impede animal movement (Davison et al. 2009, Tucker et al. 2018). Parks in city centers, backyards in suburbs, and high-density apartment blocks in inner city areas all influence habitat permeability (Cox et al. 2016) and will directly affect foraging strategies in these environments. For terrestrial species, fences, buildings, or ridges can represent barriers that can increase the cost of movement or prevent movement altogether through such environments (Shepard et al. 2013). As a result, terrestrial mammals frequently avoid them (Wall et al. 2006, Kertson et al. 2011, Hoffman and O'Riain 2012b) and often rely on human passages through environments, including transport routes, such as roads or railways, or wildlife corridors carved for conservation purposes. Although these may reduce the time and energy associated with travel (Hägerling and Ebersole 2017), they can introduce risks directly through collision with vehicles (Borkovcová et al. 2012, Murray and Clair 2015) or indirectly through exposure to predators (Fleming and Bateman 2018). Soaring birds, by contrast, have been found to use the updrafts created by buildings to engage in topographic soaring in the urban airscape (Shepard et al. 2016). Fences or flat-roof buildings also offer excellent perching opportunities for predatory birds to exploit when hunting (Chace and Walsh 2006), for colonial birds to use as nesting spaces (Maciusik et al. 2010), and for numerous species to use as refuges away from the reach of people and dogs (Chace and Walsh 2006).

The risks from road traffic (Borkovcová et al. 2012, Murray and Clair 2015) and the risks that emerge from conflict with domesticated animals (e.g., dogs; Kays and Parsons 2014) and people (Woodroffe et al. 2007, Warren 2009, Hockings et al. 2015) also affect a species's movement ecology (Gallagher et al. 2017) and can lead to reduced home ranges (Gehrt et al. 2009). Although such risks are usually predictable in space (e.g., dogs in gardens) and can be associated with specific landmarks (e.g., roads, fences), the magnitude of risk can alter dramatically with artificial and somewhat unpredictable time schedules, depending on human activities (e.g., road traffic) and human attitudes toward wildlife (Gehrt et al. 2009). This is obviously exacerbated in urban environments, and species exposed to such risks are known to adjust their behavior accordingly. For example, birds such as feral pigeons (*Columba livia*) or mockingbirds (*Mimus polygotta*) recognize humans that may represent a threat and adjust their responses accordingly (Sol et al. 2013). Commonly, this results in individuals temporarily avoiding human-modified landscapes and restraining their activity in such environments (Gehrt et al. 2009, Fehlmann et al. 2017a, Wilkie and Douglas-Hamilton 2018) but remaining in close proximity to them so that they can exploit foraging opportunities when risks are lower (Graham et al. 2009, Lewis et al. 2015, Šálek et al. 2015). Consequently, hotspots of human–wildlife conflict typically occur at the periphery of cities or farms (Kays and Parsons 2014), especially where these coincide with protected areas or refuges (Naughton Treves 1998, Woodroffe and Ginsberg 1998, Fehlmann et al. 2017b). Within the urban environments, parks can also constitute refuges from which urban-foraging species can commute (Rodewald and Gehrt 2014, Grafius et al. 2017). Therefore, maximizing the interconnectivity of natural resources for wildlife through human-altered landscapes may ease forays between refuges and human-derived resources (Michalski et al. 2006), which can increase contacts with humans and potentially conflicts. More research would be required to fully understand and predict these risks, which can jeopardize the success of conservation measures.

### Social structure

Variations in crop and urban foraging between individuals in the same social group can arise because of differences in personality, plasticity, or learning (figure 1). Variations in crop- and urban-foraging propensity might additionally cause or be caused by variations in space use and time budgets (Beckmann and Berger 2003a, Strum 2010), as summarized in the above sections. This can create challenges for the maintenance of group cohesion and synchrony (Ruckstuhl and Kokko 2002, Shannon et al. 2008, King and Cowlishaw 2009), coordination and group decision-making (Dostálková and Špinka 2007, Rands et al. 2008, Herbert-Read 2016), and, more broadly, social structures (Fichtel and Manser 2010, King et al. 2018).

Group cohesion and synchrony are known to depend on the distribution of resources and the risks in the environment. Scarce food sources and their ability to be monopolized, for example, tend to result in an increased spread of the social group (Jarman 1974, Linklater 2000, Nishikawa et al. 2014), and higher risks usually result in groups becoming more cohesive (Jarman 1974, Cowlishaw 1997, Linklater 2000). Because the risks are often higher in human-modified landscapes (Warren 2009, Hockings et al. 2015, Murray and Clair 2015) and because the relevant food sources tend to be of high quality (Hockings and McLennan 2012) and easily monopolized (Kaplan et al. 2011, Flint et al. 2016), collective decisions to exploit human-modified landscapes (as a social unit by the animals) are likely to result in significant consensus costs (Kaplan et al. 2011), borne by lower-ranked or risk-averse individuals (King et al. 2008). As a result, crop- and urban-foraging opportunities are more likely to present a constraint on group cohesion and synchrony and to cause fission and fusion of groups when specific individuals leave the group to forage in human-changed landscapes (Warren et al. 2007, Graham et al. 2009).

At present, we don't properly understand the consequences of reduced cohesion and fission for social groups using human-changed spaces, but we hypothesize that those crop- and urban-foraging individuals that tend to be risk-prone phenotypes (Michl et al. 2000, King et al. 2013) prioritize the exploitation of these new opportunities over social attraction. In the collective behavior literature, individuals’ prioritization of their own travel direction over social attraction has been termed *leading* according to need or social indifference (Conradt et al. 2009). In such cases, individuals increase their influence on group movement by increasing their assertiveness and decreasing their social attraction range. This has been shown by empirical research with shoaling fish (Ioannou et al. 2015). Where animals favor goal-oriented motion over their tendency to be social, their groups are predicted to fission (Conradt et al. 2009, Sueur et al. 2011). Although we are not aware of any systematic studies on whether and how such fission–fusion dynamics may occur because of crop and urban foraging, in the Cape Peninsula baboons, splinter troops (smaller groups that fission from a large group) are a priority for management, because they drive exceptional levels of urban raiding. Proper understanding of these processes will be particularly important for management policies aimed at removing specific “problem” individuals or groups (Swan et al. 2017), and more research is therefore needed in order to clarify these socioecological processes.

### Life history traits and population dynamics

Because individuals acquire energy faster and with more certainty in human-altered landscapes, these individuals tend to show improved body condition (Beckmann and Berger 2003b, Otali and Gilchrist 2004, Chiyo et al. 2011). This, in turn, has a positive influence on individual growth and fitness (Beckmann and Berger 2003b, Chiyo et al. 2011, Rotics et al. 2017). This is particularly true for species defined as energy maximizers, whose fitness is directly related to energy intake (Schoener 1971). In elephants (box 2), for example, crop-foraging males are larger (and potentially mature faster), which can lead to a longer breeding life span (Chiyo et al. 2011). For time minimizers, the time freed by faster foraging bouts can also be directly reallocated to finding mates, maximizing individual fitness. This, coupled with less ranging effort and smaller home ranges, allows for general population densities to increase drastically. Such phenomena have been observed in the periphery of human settlements, particularly in medium size carnivores (Chace and Walsh 2006, Yirga et al. 2013, Šálek et al. 2015), but these observations should be nuanced. In urban environments, higher densities may occur only in green spaces in which urban individuals may be condensed (Rodewald and Gehrt 2014). Indeed, although foraging opportunities might be plenty, other resources such as nesting sites may be limited and may become a limiting factor in the urban matrix (Charter et al. 2016, Hernández-Brito et al. 2018).

**Box 2. Nocturnal field trips: The case of elephants.**

As humans encroach into natural areas, African elephant (Loxodonta africana) populations encounter human settlements and fields more frequently, which often leads to chronic conflicts (Osborn 2004, Graham et al. 2009). Elephants frequently target crops at specific times of the year: when they are the most palatable (Chiyo et al. 2005, Jackson et al. 2008), when the quality of natural forage decreases (Osborn 2004), or when seasonal migratory routes take them past agricultural areas (Adams et al. 2017). When crops are mature, this foraging opportunity can provide elephants with dense, high calorie and easy to process food items (Osborn 2004). Elephants can be classified as energy maximizers (Hixon 1982), and the crop-foraging individuals, which are most frequently males (Jackson et al. 2008, Ahlering et al. 2011, Chiyo et al. 2012), tend to be bigger and benefit from a prolonged musth phase (Chiyo et al. 2011). Male biased raiding in elephants is likely to be explained by their general boldness (Sitati et al. 2003), their independent ranging behavior, and their size and strength (Shannon et al. 2008), which can allow them to more easily break through physical barriers (e.g., fences). High nutrient intake allows males to allocate more time and effort to secure mating opportunities, through increased walking and reduced time devoted to resting (Shannon et al. 2008). Overall, crop foraging might therefore have positive effects on biological fitness and population size may increase with the large amount of high-quality forage that crops make available to them (Mramba et al. 2019). However, because elephant–human conflicts frequently result in fatalities (Kioko et al. 2008), crop foraging can have severe repercussions on elephant population dynamics and can create an ecological trap. The high risks associated with encountering humans result in appropriate (adaptive) behavioral responses from the elephants, typically avoiding human settlements (Pozo et al. 2018), moving faster where there are higher chances of conflicts (Graham et al. 2009), predominantly at night (Sitati et al. 2003, Graham et al. 2009, Wilkie and Douglas-Hamilton 2018), and preferentially crop foraging close to refuge areas (Jackson et al. 2008).

**Figure:**
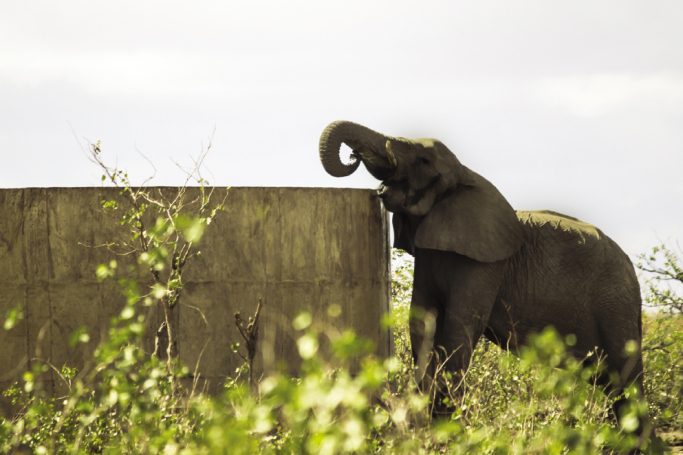
Loxodonta africana. Photograph: Gaelle Fehlmann.

So far, we have generally portrayed anthropogenic food as high-energy, predictable food sources, but they can vary hugely in nutritional value and availability. Food refuse or discards, for example, although they are readily accessible, can be nutrient and protein deficient and temporarily unavailable (e.g., after refuse collection; Grémillet et al. 2008, Murray et al. 2015); contain nonfood items that can be ingested, such as plastic or rubber bands (Henry et al. 2011); and pose health risks because of pathogens (Drewe et al. 2012, Serieys et al. 2019a). Bin or landfill foraging can therefore be associated with reduced body condition, as has been reported for gannets (Grémillet et al. 2008) and coyotes (Murray et al. 2015). However, it is difficult to disentangle cause and effect in these data—whether frequent raiding reduces individual condition or whether low-quality individuals preferentially source human-derived food items (Murray et al. 2015). Another hypothesis could be that the abundance and predictability of food items, coupled with reduced predation risk in human-altered landscape, allow for the survival of lesser quality individuals resulting in larger population densities but composed of many low quality individuals and a handful of winners (Shochat 2004). More research is clearly needed to disentangle the positive and negative impacts that human-derived food has for individuals at different life stages (Duhem et al. 2005, Grémillet et al. 2008). Identifying the situations in which individuals are worse off by foraging on crops or in cities is important, because it allows us to define the potential ecological traps that may arise from crop- or urban-foraging behaviors (Šálek et al. 2015), and result in drastic population decreases, particularly in species whose reproductive strategies are particularly slow (Hockings et al. 2015).

### Community dynamics

We have reviewed the consequences of crop and urban foraging on several aspects of biology and although there is a general positive impact on individual's fitness and population dynamics (figure 1), these can also have broader consequences at a community level. These can alter trophic cascades, particularly if the exploitation of novel foods results in reduced predation on natural prey or if the increased population density leads to the overexploitation of an ecological niche (Hebblewhite et al. 2005, Jones et al. 2016). In fact, the increased population density of species thriving in human-altered landscape is also likely to affect competitors. For example, kleptoparasitism by hyenas is known to challenge wild dogs’ survival because of the costs of losing a prey item (Gorman et al. 1998). As hyenas’ density increases, it is likely that these dynamics may be reinforced and cause other sympatric carnivores such as wild dog and cheetah to decrease (Green et al. 2019). In addition, other ecological services provided by these crop or urban foraging species would be affected. For example, reduced movement through natural landscapes can reduce the dissemination of seeds (Chapman and Onderdonk 1998, Naoe et al. 2016, Sebastián-González et al. 2019) or the maintenance of open habitats or corridors (Cumming et al. 1997). Decreased interactions between species is then likely to increase fragmentation of resources and habitats (Berger-Tal and Saltz 2019). In human-altered landscapes, the presence and increased movement of wild species can increase the spread of exotic and invasive species (Mellado and Zamora 2014) or diseases to humans or domestic species (Daszak et al. 2000, Donnelly et al. 2006, Flint et al. 2016). These challenges will need to be met with urgency by conservation biologists and ecologists.

Globally, disease transmission between humans and wildlife is occurring at an increasing rate, posing a substantial global threat to public health and biodiversity conservation (Jones et al. 2008). Human activities linked to urban and rural land use can interact with disease agents (May 1988) and may result in the alteration of parasite transmission rates, host range, and virulence (Daszak et al. 2000, Patz et al. 2000). Together, these changes may pose a significant conservation risk to wildlife populations living at the urban edge (Serieys et al. 2019b) and particularly to nonhuman primate populations (Wallis and Rick Lee 1999, Drewe et al. 2012, Olarinmoye et al. 2017). With higher population density, which is of central importance to infection rates in the hosts of directly transmitted parasites, the risks are multiplied (Vitone et al. 2004, Silk et al. 2019). Close human contact may also drive higher parasite prevalence in wildlife, notably in baboons (Ghandour et al. 1995, Müller-Graf et al. 1996, Munene et al. 1998), whereas anthropogenic disturbances, such as logging and forest fragmentation, have been shown to alter the dynamics of parasite infection in primate populations (Chapman et al. 2005, Gillespie et al. 2005). Identifying general principles governing disease occurrence is critical for managing vulnerable wildlife populations and mitigating risks to human and animal health. However, it is currently unclear what aspects of anthropogenic changes to the physical environment facilitate the transmission of infectious agents among wildlife and humans (Hassell et al. 2017). The development of improved conservation strategies to deal with established and changing patterns of disease demands that we increase our efforts to understand the interplay between the alteration of ecosystems and disease transmission probabilities.

## The challenge of managing wildlife foraging in human-modified landscapes

A change of societal values in developed countries has led to a greater tolerance toward wildlife, and this tolerance and desire for coexistence—rather than conflict—may explain why some species are currently colonizing or recolonizing human-altered landscapes (Carter and Linnell 2016, Bruskotter et al. 2017). Despite these changes, conflicts between wildlife and people emerge as a result of negative interaction that can cause economic loss (e.g., crop depredation or damage to properties; Ogada et al. 2003), disruptive behaviors (e.g., dissemination of trash when foraging on discards Belant 1997, Kaplan et al. 2011, Flint et al. 2016), or increased anxiety (e.g., when the encounter of the wild species result in a risk for humans; Beamish 2009, Lewis et al. 2015, Acharya et al. 2016). In all these ways, conflicts can affect people's perception of biodiversity, hinder conservation goals and ultimately create tensions among people themselves (Chan et al. 2007, Barua et al. 2012).

To mitigate human–wildlife conflict across the globe, people have traditionally inserted barriers in the landscape (table 2; Woodroffe et al. 2007), actively chased wildlife away (table 2; Ogada et al. 2003, Warren 2009), or selectively removed individuals, groups, or populations of species that pose a chronic threat (table 2; Donnelly et al. 2006, Katsvanga et al. 2006). Less frequently, individuals or entire groups may be relocated (Swan et al. 2017) to areas where the source of conflict is absent. These methods are still widely used today and vary in their effectiveness, scalability, and level of public acceptability. Shepherding and fencing livestock, for example, are widely supported as effective nonlethal methods for reducing conflicts with carnivores such as lions (*Panthera leo*), leopards (*Panthera pardus*), and cheetahs (*Acinonyx jubatus*; Woodroffe et al. 2007, Hayward and Kerley 2009). However, fencing may have limited success with burrowing and climbing species (Kioko et al. 2008, Osipova et al. 2018) and can have devastating effects on migratory species such as antelopes (Harris et al. 2009, Hayward and Kerley 2009). Culling or harvesting is often not realistic because of the risk it poses to the viability and demography of local populations (Milner et al. 2007) and the growing opposition from the general public to indiscriminate lethal methods (Shivik et al. 2003, Treves and Naughton-Treves 2005, Slagle et al. 2017). Guarding has been shown to significantly reduce conflicts but is costly to sustain by professionals (table 2; Woodroffe et al. 2007a, Davies et al. 2011) and may lead to social inequalities and hinder people's well-being. For example, crop guarding by children in rural communities hinders school attendance (Barua et al. 2012). Other methods may involve the use of repellents (table 2; Hill and Wallace 2012, Zarco-Gonzalez and Monroy-Vilchis 2014) and supplementary feeding patches (table 2; Oro et al. 2008, Kaplan et al. 2011), but none has yet proven effective in the long term.

**Table 2.** Overview of potential management strategies.

	Method	Examples	Limitations	Costs	Labor	Durability	Precision	Public acceptability	Refs
Nonlethal deterrents	Disruptive	Fencing	Cost	+ + +	+	+ +	—-	+ +	1–3
		Repellents or fladry	Habituation	-	+	-	+	-	4–6
		Field ranger	Costs, human flaws	+ +	+ + +	+ +	+ + +	+ +	7–9
	Population control	Translocation	Moving the problem	+ +	-	+ +	+ + +	-	10
		Contraception or sterilization	Labor intensive	+ +	+ + +	-	+ + +	-	11
	Aversive	Conditioned taste aversion	Labor intensive	-	+ + +	+ +	+ + +	-	12
Nonlethal attractants	Provisioning	Supplemental feeding patch	Increased feeding	+	+	-	—-	+ +	13–14
Lethal	Population control	Removal of individuals or groups	Identifying problem individuals	+ +	+	- -	+ + +	+ +	15–17
				++	+	- -	-	+ + +	

*Note:* Considerations and positive (+) and negative (-) outcomes associated with the various aspects or processes related to the management strategies. References cited: 1–3. Woodroffe et al. 2007, Hayward and Kerley 2009, Osipova et al. 2018, 4–6., 7–9. Ogada et al. 2003, Warren 2009, Fehlmann et al. 2017b, 10–12. Swan et al. 2017, Snijders et al. 2019, 11. Massei and Cowan 2014, 12. Snijders et al. 2019, 13–14. Kaplan et al. 2011, 15–17. Treves and Naughton-Treves 2005, Milner et al. 2007, Swan et al. 2017.

Selecting the correct strategy to manage the impact of crop- and urban-foraging wildlife and the conflicts that emerge from this behavior (human–wildlife and among people) is critical, because an inappropriate or ineffective management strategy may result in an arms race between humans and crop- or urban-foraging species (Ogada et al. 2003, Davies et al. 2011, Kaplan et al. 2011). When conflicts emerge, we suggest a focus on simultaneously raising the costs and reducing the rewards related to these foraging behaviors. This might be particularly challenging because the time and energy constraints of populations foraging on human-derived foods are relaxed, and the additional time and energy available may potentially outweigh failed foraging attempts and exacerbate natural behavioral plasticity (figure 1). Specifically, additional time and energy available to crop- and urban-foraging individuals can potentially reduce the efficacy of fences and guarding methods used in isolation of other methods. We therefore believe there is no single or simple solution. Instead, management strategies require proper understanding of the biological problems and development of bespoke solutions. Indeed, there exists the need for a two-pronged response to human–wildlife conflict (table 1), where scientific studies accurately measure the extent of wildlife damage and how biological factors influence this, whereas social studies look to examine the human component of the conflict, taking into account the varying views of directly affected parties as well as third parties and the prevailing socioeconomic environment. Collaboration between these two endeavors will ensure that mitigation strategies have the best chance of success (Atwood and Breck 2012, Carter and Linnell 2016).

## Conclusions

Human activities now dominate the natural environment and most species encounter human-altered features on a daily basis. Although most of these changes have adverse impacts on wildlife communities, a growing number of studies around the world provide empirical evidence for the successes of species foraging in human-altered environments (Chace and Walsh 2006, Hockings et al. 2015, Fleming and Bateman 2018). Species characterized by phenotypic plasticity and a generalist diet appear well adapted to exploit human-modified environments (Griffin et al. 2017). Furthermore, long lived species that live in socially complex groups and possess higher cognitive skills are likely to learn to successfully exploit human-derived food in croplands and urban areas (Barrett et al. 2018). In the present article, we proposed that foraging in human-modified landscapes not only results in a rapid dietary change but presents a novel selective pressure that may lead to important changes in key behavioral traits such as movement patterns, activity, and energy budgets to social dynamics within groups and life history traits. We demonstrated how these aspects of species biology are intertwined, resulting in new population and community dynamics and challenges. We highlighted how these changes can affect management of these individuals, populations and species in conflict with humans in altered environments. How changes in these traits influence reproductive strategies and mate choice remains an important area for future research, which will influence both population dynamics and the spread of such behaviors in populations.

Understanding how wildlife species respond to novel selection pressures in the Anthropocene is important from both a biological and wildlife management perspective. Devising novel approaches and methods to ensuring that more wildlife has better welfare and conservation status within and adjacent to urban and rural landscapes is highlighted as a critical future goal. Realizing this goal will require that we continue to innovate when seeking to understand the drivers of human–wildlife conflict and its mitigation while continuing to develop a theoretical framework for understanding responses of wildlife to diverse global human-modified landscapes.

## Author Biographical

Gaelle Fehlmann (fehlmanng@gmail.com) is a postdoctoral fellow at the Max Planck Institute for Animal Behavior, in Bodensee, Germany. Justin O'Riain is a professor and director of the Institute for Communities and Wildlife in Africa, in the Department of Biological Sciences at the University of Cape Town, in Cape Town, South Africa. Ines Fürtbauer is a senior lecturer and head of the Behavioural Ecology and Endocrinology Laboratory and Andrew King is an associate professor and head of the SHOAL group in the Department of Biosciences at Swansea University, in Swansea, Wales, in the United Kingdom.
